# Effects of hydrodynamic cavitation combined with snail enzyme treatment on the structure and functional properties of water-soluble dietary fiber in rice husks

**DOI:** 10.1016/j.ultsonch.2025.107236

**Published:** 2025-01-19

**Authors:** Zhigang Quan, Mingming Chen, Dongjie Zhang

**Affiliations:** a College of Food Science, Heilongjiang Bayi Agricultural University, Daqing 163319, PR China; b National Coarse Cereals Engineering Research Center, Daqing 163319, PR China; c Key Laboratory of Agro-Products Processing and Quality Safety of Heilongjiang Province, Daqing 163319, PR China

**Keywords:** Rice husk, Hydrodynamic cavitation, Snail enzyme, Water-soluble dietary fiber

## Abstract

In this study, we adopted the synergistic modification technology of hydrodynamic cavitation and snail enzyme, to improve the yield and activity of soluble dietary fibers (SDFs) of rice husk. The physicochemical properties, structural changes, and inhibition of α-glucosidase and α-amylase of SDFs were examined *in vitro*. This synergistic treatment significantly increased the yield of SDFs to 18.64 % ± 0.16 %, significantly reduced the particle size to 122.33 ± 0.26 nm, and significantly increased the specific surface area to 1.718 ± 0.002 m^2^/g. The absolute value of the zeta potential significantly increased to −36.39 ± 0.12 mV, indicating an excellent solution stability and gel-forming ability. At the same time, the water-holding, oil-holding, and swelling capacities were significantly enhanced, reaching 8.52 ± 0.09 g/g, 4.85 ± 0.29 g/g, and 7.29 ± 0.25 mL/g, respectively. Structural analysis showed that the synergistic treatment destroyed the fiber structure, produced a large number of small molecule fragments, and significantly changed the monosaccharide components and functional group distribution. Functional evaluation showed that the inhibitory effect of CE-SDF on α-glucosidase and α-amylase was significantly enhanced, and enzymatic reaction kinetic analysis revealed that both enzymes were competitive inhibitors, with IC_50_ values of 2.893 and 1.727 mg/mL, respectively. In summary, the synergistic modification of hydrodynamic cavitation and snail enzyme greatly optimized the structural and functional properties of rice husk SDFs, providing a theoretical basis for its application in the field of hypoglycemic drugs and functional foods.

## 1 Introduction

Rice is one of the main nutritional sources worldwide, particularly in Asia. In 2003, the global rice production reached 741 million tons, of which, approximately 148 million tons was rice husks (∼20 % of the grain weight) [1]. Rice husk is usually discarded as biological waste, despite its high content of abundant dietary fiber (DF), which is a complex mixture of lignin, hemicellulose, cellulose, and silica [2], [3]. Rice husk has attracted increasing attention owing to its low cost, low environmental impact, water insolubility, chemical stability, high mechanical strength, and high usability. In addition, in 2013, the US Food and Drug Administration classified rice husk DF as Generally Recognized as Safe (GRAS), indicating that it can be employed as a food ingredient. Importantly, rice husk possesses various biological and nutritional properties, including antimicrobial, anti-allergic, and anti-inflammatory activities [4], [5].

DF is regarded as the “seventh most important nutrient” [6], in addition to being classified as a functional food ingredient with broad application prospects. This is of particular importance considering the recent increasing focus of consumers on healthy eating habits [7]. DF can be divided into two categories, namely soluble dietary fiber (SDF) and insoluble dietary fiber (IDF). Examples of SDFs include inulin, pectin, and β-glucan, which exhibit excellent biological properties because of their high water solubilities. Further, it has been reported that SDFs demonstrate strong antioxidant activities in addition to other functional effects, including the reduction of the blood glucose response and regulation of the blood cholesterol levels [8]. Chen et al. [9] showed that SDF significantly improved the blood glucose levels, insulin resistance, and metabolism of patients with type 2 diabetes during a short-term intervention phase. Furthermore, several studies have reported mechanisms via which SDF reduces postprandial blood sugar, such as slowing down gastric emptying [10], promoting insulin secretion, antagonizing glucagon [11], inhibiting starch hydrolysis [12], and inhibiting small intestinal glucose absorption [13]. Compared with IDFs, SDFs exhibit more desirable textures and tastes, and they are more convenient for use in food processing applications [14]. Furthermore, SDFs exhibit superior emulsifying properties than IDFs for gel formation [15], whilst also imparting systems with increased viscosities [16]. In contrast, when added to foods, IDFs (e.g., cellulose, lignin, and hemicellulose) can have adverse effects in terms of the texture, taste, and flavor. Dense IDFs endow cell walls with significant mechanical strength, rendering their degradation challenging by enzymatic, chemical, or other approaches. Consequently, SDFs exhibit a higher application value than IDFs. Therefore, an efficient approach based on the combination of two or more methods for obtaining higher SDF yields from rice husk as well as more desirable characteristics is highly needed.

Several SDF modification procedures exist, among which, the physical and enzymatic methods have attracted widespread attention owing to their environmental friendliness, high efficiency, mild operation, and low cost. The hydrodynamic cavitation technology is a physical method with great application potential. As an advanced processing tool, it has been widely used in many fields [17], such as water jet cutting devices and engine fuel injection systems [18]. Hydrodynamic cavitation technology is a highly efficient, low-energy and environmentally friendly wastewater and sludge pretreatment technology with the advantages of simple equipment, low operating cost and suitability for industrial scale. It generates high temperature, high pressure and ·OH radicals through bubble collapse, which significantly improves the biodegradability index of wastewater (BOD_5_/COD) and sludge dissolved organic matter (SCOD), and enhances biodegradation efficiency. Studies have shown that hydrodynamic cavitation combined with advanced oxidation processes (such as Fenton reagent, ozone, and hydrogen peroxide) can further improve the degradation of pollutants. At the same time, optimizing the design and operating conditions of equipment such as Venturi tubes can effectively reduce energy consumption, showing great potential for industrial applications [19], [20], [21]. The method is based on the principle that when a fluid is forced through a nozzle at high velocity, a cavity is spontaneously formed within the fluid. The cavity expands and contracts rapidly, producing intense localized jets of temperature, pressure, and velocity. The hydrodynamic cavitation treatment causes the large particles in the fluid to break down into smaller particles due to the intense destructive forces generated [22]. Hydrodynamic cavitation technology is an innovative material modification technology that enhances the erosion effect of the jet through the high-intensity impact force released by cavitation bursting, thereby effectively transforming the material structure and significantly optimizing the product characteristics [23]. Hydrodynamic cavitation is widely used in the cell disruption of microorganisms and microalgae [24], [25], as well as for the recovery of intracellular enzymes [26]. Hydrodynamic cavitation, as an emerging food processing technology, has been successfully applied in the field of DF modification and has achieved remarkable results. Wu et al. [27] and Tian et al. [28] reveled that the cavitation jet technology can effectively modify DF in okara, improving the physical and chemical properties and thickening ability of DF by significantly changing its spatial structure. In addition, the enzymatic method has become a highly respected processing method owing to its strong specificity, gentle operation, high efficiency, stability, and environmental friendliness. Single enzymes such as cellulase, xylanase, and pectinase can only act on specific polysaccharides. In modification treatments where multiple enzymes are combined, problems such as complex modification processes, cumbersome operations, and low efficiency often occur, due to the different enzymes requiring different optimal conditions. Snail enzyme is a natural complex enzyme derived from snail capsules and digestive tracts. It contains more than 20 enzymes such as cellulase, pectinase, amylase, and protease. It has the ability to efficiently degrade a variety of insoluble DFs and has attracted increasing attention owing to its natural origin, low cost, and abundancy. Therefore, modification with snail enzyme is a promising method for modifying DF [29], [34], [30], [31]. In addition, snail enzymes have been widely used in the disruption of yeast cell walls owing to their powerful digestive ability [32]. However, DF modification via hydrodynamic cavitation combined with snail enzymes is understudied. In this study, SDF was extracted from rice husk via hydrodynamic cavitation, the snail enzyme method, and a combination of both methods, and its yield, structural characteristics, and functional properties were systematically measured and characterized. We aimed to explore the most efficient extraction method affording high-quality SDF and provide a scientific basis and technical support for in-depth understanding of the potential value of rice husk resources and their wide application in the food industry.

With these considerations in mind, we report the modification of rice husk SDF using hydrodynamic cavitation combined with the snail enzyme method. The physicochemical properties and structural changes of rice husk SDF before and after modification are evaluated and compared, and the inhibitory effects of the resulting modified SDFs on the *in vitro* activities of α-glucosidase and α-amylase are evaluated. This study aims to lay the foundations for the further development and utilization of rice husks through improvement of their SDF functional components.

## 2 Materials and methods

### 2.1 Materials and reagents

Defatted rice husk (moisture content 3.84 %) was supplied by Harbin Wuchang Rice Co., Ltd. (Harbin, China). High-temperature-resistant α-amylase (4 × 10^4^ U/mL), neutral protease (6 × 10^4^ U/mg), amyloglucosidase (10 × 10^4^ U/mg), snail enzyme, α-glucosidase, and p-nitrophenol glucopyranoside (pNPG) were obtained from Sigma Aldrich (St. Louis, MO, USA). Bovine serum albumin was obtained from Aladdin, Chemical Co. (Shanghai, China). All other chemicals and reagents were of analytical grade.

### 2.2 SDF preparation

SDF was prepared from the rice husk powder as described by Quan et al. [33], with slight modifications. More specifically, rice husk powder (5.0 g) was crushed through a 60-mesh sieve and added to the phosphate buffer in a powder-to-liquid ratio of 1:40 (g/mL). After adjusting the pH to 6.0 using 1 M HCl and 1 M NaOH, high temperature-resistant α-amylase (1 %) was added, and the mixture was incubated with the GDE Enzymatic Digester (VELP Scientifica, Usmate, Italy) for 20 min in a water bath at 95–100 ℃. The pH was then adjusted to 7.0, and a neutral protease solution (1 %) was added to the mixture, which was maintained at 60 ℃ for 30 min. Subsequently, the pH was adjusted to 4.5, amyloglucosidase (1 %) was added, and the mixture was incubated at 60 ℃ for 30 min. The enzyme was inactivated at 100 ℃ for 10 min. The digest was then filtered using a CSF6 filter unit, and the filtrate was centrifuged (4000 rpm, 20 min) and concentrated to 1/4–1/5 of the original solution volume. After precipitation in ethanol (95 %, v/v), the precipitate was freeze dried at −108 ℃ to obtain the sample denoted as non-processed soluble dietary fiber (N-SDF).

### 2.3 SDF modification

#### 2.3.1 Snail enzymatic hydrolysis

Snail enzymatic hydrolysis was performed as described by Hassan et al. (2019), with slight modifications. N-SDF (4.0 g) was added to distilled water in a solid-to-liquid ratio of 1:40 (g/mL), and the pH was adjusted to 5.5. Snail enzyme (2 % w/w) was added to the sample solution, and enzymatic hydrolysis was performed at 40 ℃ for 4 h, followed by enzyme inactivation at 95 ℃ for 5 min. Subsequently, following the SDF preparation procedure described in Section 2.2, the enzymatically-treated soluble dietary fiber (E-SDF) was obtained via freeze drying.

#### 2.3.2 Hydrodynamic cavitation

**Fig. 1:**
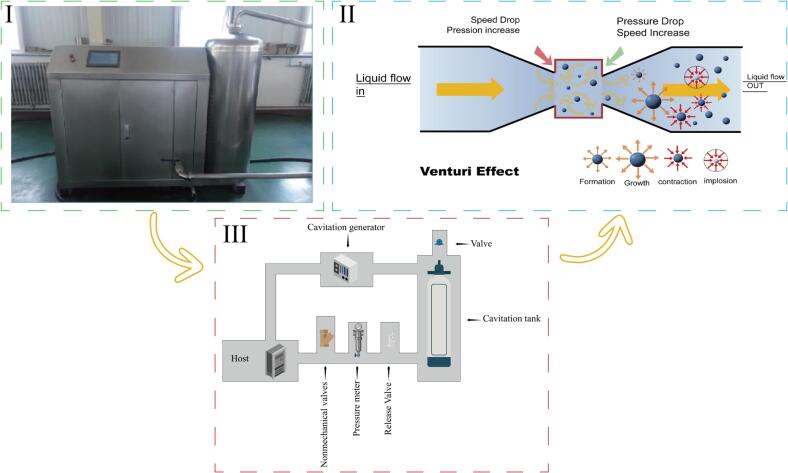
Pictures and schematic diagram of hydrodynamic cavitation device. (The system components include electronic control system, cavitation tank, and tank body; SL-1000 hydrodynamic cavitation equipment parameters: voltage (380 V), power (135 KW), dimensions (2600*2800*1680 mm)).

Hydrodynamic cavitation was performed according to the procedure described by Tian et al. [35], with slight modifications. Rice husk was pre-processed using a mechanical crusher, followed by sieving (100–mesh). The screened rice husk powder was then added to distilled water in a solid-to-liquid ratio of 1:30 (g/mL) and stirred thoroughly. As shown in Fig. 1, the resulting mixture was poured into a hydrodynamic cavitation reactor (SL-1000, Zhongsen Huijia Technology Development Co., Ltd., Beijing) for 15 min in a water bath under 0.4 MPa and 25 ℃; subsequently, the sample solution was collected and cooled. Following the SDF preparation procedure described in Section 2.2, the hydrodynamic cavitation-treated soluble dietary fiber (C-SDF) was obtained via freeze drying.

#### 2.3.3 Hydrodynamic cavitation combined with snail enzyme treatment

Hydrodynamic cavitation was combined with snail enzyme treatment and performed as described in Section 2.3.2, with slight modifications. The E-SDF liquid sample obtained by hydrodynamic cavitation treatment (Section 2.3.1) was subjected to enzymatic treatment. The obtained product was freeze dried and denoted as CE-SDF.

### 2.4 Component analysis

The SDF and IDF contents were determined based on the procedure described in AOAC 991.43 Official Method [36], and the moisture and ash contents were determined separately following AOAC 925.40 (2005). The protein contents of the samples were determined using the Kjeldahl method (AOAC 925.40, 2000), while the fat contents of the samples were determined using the Soxhlet extraction method (AOAC 920.39).

### 2.5 Structural analysis

#### 2.5.1 Transmission electron microscopy

Transmission electron microscopy (TEM) was performed as described by Deng et al. [37], with slight modifications. After fixing the water-cavitation-treated and untreated rice husk powder samples with 2.5 % glutaraldehyde, the samples were rinsed with 0.1 M phosphate buffer at pH 7.0. Subsequently, the samples were fixed with a 1 % osmium solution and dehydrated with ethanol (30 %–100 %) and pure acetone. After infiltration with the embedding agent, the samples were heat-embed at 70 ℃ and sliced to 70–90 nm. The slices were stained with lead acetate and uranyl acetate and then observed using TEM (Hitachi-HT7800, Hitachi).

#### 2.5.2 Scanning electron microscopy

The effects of the different treatment methods on the SDF microstructure were evaluated using scanning electron microscopy (SEM; Zeiss Gemini Sigma 300, Zeiss Merlin Co., Germany). The SDF sample was fixed on a double-sided tape, and a thin gold layer was sputtered under vacuum for 60 s. The images were then collected at an acceleration voltage of 3.0 kV under 30×, 75×, 300×, and 400× magnification.

#### 2.5.3 Small-angle X-ray scattering

To perform the small-angle X-ray scattering (SAXS, Anton Paar SAXSess MC2, Xenocs Co., France) experiments, 20 mg of the SDF sample was loaded into the paste cell, sealed, and placed in the instrument sample holder. After the vacuum reached 5–10 Pa, the SAXS measurements were performed at 25 ℃ over a period of 40 min.

#### 2.5.4 Fourier transform infrared spectroscopy

Fourier transform infrared spectroscopy (FT-IR) was employed (Nicolet iZ-10, Thermo Nicolet, USA) to evaluate the effects of the various treatment methods on the SDF functional groups. The SDF sample was mixed with potassium bromide powder in a ratio of 1:100, ground thoroughly, and pressed into tablets. FT-IR was performed over a scan range of 4000–400 cm^−1^ over 64 scans with a resolution of 4 cm^−1^.

#### 2.5.5 High-performance liquid chromatography

The monosaccharide compositions of the various SDF samples were separated and identified using high-performance liquid chromatography (HPLC, Agilent 1100, Shimadzu Co., Japan). The SDF solution (100 μL, 4–5 g/L) was mixed with 100 μL of 4 mol/L trifluoroacetic acid (TFA) and the air was replaced with N_2_. After sealing the tube, hydrolysis was carried out at 110 ℃ for 2 h. After cooling, the residual TFA was removed by adding 200 μL of methanol, followed by drying with nitrogen gas; this process was repeated in triplicate. Subsequently, a 0.3 mol/L NaOH solution (50 μL) and a 0.5 mol/L solution of 1-phenyl-3-methyl-5-pyrazolone (PMP) in methanol (50 μL) were added. After mixing, the mixture was reacted at 70 ℃ for 100 min, cooled, derivatized, and injected for analysis.

#### 2.5.6 Gel permeation chromatography

Gel permeation chromatography (GPC) was performed to determine the molecular weights of the SDF samples. The sample (50 mg) was dissolved in a mobile phase (0.1 mol/L NaNO_3_ + 0.06 % NaN_3_, aqueous solution) and diluted to volume. The analysis was performed using a flow rate of 0.5 mL/min, a column temperature of 40 ℃, and a sample injection volume of 20 μL (LC20 + RD20A Shimadzu Corporation, Japan; Ultrahydrogel TMLinear column, 300 mm × 7.8 mm).

#### 2.5.7 Nuclear magnetic resonance spectroscopy

Nuclear magnetic resonance (NMR) spectroscopy was performed as described by Wang et al. (2019), with slight modifications. The samples were dissolved in D_2_O and shaken thoroughly to ensure complete dissolution. A Bruker AVANCE NEO 500 M Spectrometer System (Bruker, Rheinstetten, Germany) was used for analysis, and the corresponding ^1^H and ^13^C NMR spectra were recorded at 25 ℃ and 500 MHz.

#### 2.5.8 Particle size distribution

Each sample (1 mg) was placed in a 5-mL centrifuge tube together with water (2 mL), followed by ultrasonication for 5 min. The supernatant (1 mL) was added slowly to the sample cell to prevent bubble formation, and the particle size distribution was analyzed using a Malvern laser particle analyzer (Zetasizer Nano ZS90, Malvern, UK). After preheating the instrument for 30 min, water/ethanol was selected as the solvent and the samples were preheated to 25 ℃ and measured in triplicate.

### 2.6 Physical and chemical properties and functional characteristics of the samples

#### 2.6.1 Water-holding capacity

The SDF sample (0.5 g) was dissolved in distilled water (25 mL), allowed to stand at 25 ℃ for 24 h, and then subjected to centrifugation at 4000 rpm for 20 min. After 20 min, the precipitate was collected and weighed. The water-holding capacity (WHC) was calculated as:

(1) $$\mathrm{WHC}\left(\text{g}/\text{g}\right)=\frac{\text{m}1-{\text{m}}_{0}}{\text{m0}}$$

where m_1_ and m_0_ represent the mass of the precipitate (g) and mass of the original SDF (g), respectively.

#### 2.6.2 Oil-holding capacity

The SDF sample (0.5 g) was dissolved in soybean oil (30 mL) and allowed to stand at 25 ℃ for 3 h. After centrifugation at 4800 rpm for 10 min, the precipitate was collected and weighed. The oil-holding capacity (OHC) was calculated as:

(2) $$\mathrm{OHC}\left(\text{g}/\text{g}\right)=\frac{\text{W}0}{\text{WS}}$$

where W_0_ and W_s_ represent the mass of the oil-adsorbed sample (g) and mass of the original SDF (g), respectively.

#### 2.6.3 Swelling capacity

Each sample (1 g) was accurately weighed in a graduated test tube. Distilled water (30 mL) was added and after shaking, the sample was allowed to set at 25 ℃ for 12 h. The swelling capacity (SC) was calculated as:

(3) $$\mathrm{SC}\left(\text{mL}/\text{g}\right)=\frac{{\text{A}}_{2}-{\text{A}}_{1}}{\text{m}}$$

where A_1_, A_2_, and m represent the sample volume before water absorption (mL), sample volume after water absorption (mL), and mass of the original SDF (g), respectively.

#### 2.6.4 Determination of the *in vitro* enzyme inhibition activity

##### 2.6.4.1 Inhibition of α-glucosidase

To determine the α-glucosidase inhibition activities of the SDF samples, the method described by Yang et al. [39] was employed, with slight modifications. A 0.5 U/mL α-glucosidase solution was prepared using a 0.1 M phosphate buffer solution (pH 6.8), and a pNPG solution was prepared at a concentration of 2.5 mM. To prepare the samples for analysis, the desired sample (40 μL) was added to a 96-well plate with the pNPG solution (50 μL), and reacted at 37 ℃ for 15 min. Subsequently, the α-glucosidase solution (80 μL) was added, and the reaction was continued at 37 ℃ for 30 min. Finally, a 0.2 mol/L Na_2_CO_3_ solution (50 μL) was added to terminate the reaction. To prepare the blank sample, the enzyme solution was replaced with an equal volume of the buffer solution. To prepare the control sample, the sample was replaced with an equal volume of double distilled water (ddH_2_O). For the blank control, the sample was replaced with an equal volume of ddH_2_O and the enzyme solution in the buffer. For each system, the absorbance was determined at 405 nm using a microplate reader (SPECTROstar Nano, BMG LABTECH Co., Germany). Acarbose was used as a positive control. The enzyme inhibition rate was calculated as:

(4) $$\alpha -glucosidase\;inhibition\;rate=1-\frac{A_{\mathrm{sample}}-A_{\mathrm{sampleblank}}}{A_{\mathrm{control}}-A_{\mathrm{controlblank}}}\times 100\%,$$

where A_sample_, A_sample blank_, A_control_*,* and A_control blank_ represent the absorbance of the experimental group, absorbance of the blank, absorbance of the control, and absorbance of the blank control, respectively.

##### 2.6.4.2 Dynamic analysis of α-glucosidase inhibition

To evaluate the α-glucosidase inhibition activities of the SDF samples, the methods of Ullah et al. [40] and Zheng et al. [41] were followed with slight modifications. The relevant dynamic parameters for α-glucosidase inhibition were determined for the various samples. Initially, using an α-glucosidase concentration of 0.5 U/mL, the sample solution (40 μL; different concentrations) was added to the 96-well plate along with pNPG (50 μL). After heating at 37 ℃ for 15 min, the α-glucosidase solution (80 μL) was added, and the reaction was continued at 37 ℃ for 30 min. After this time, a 0.2 mol/L Na_2_CO_3_ solution (50 μL) was added to terminate the reaction. The absorbance was measured at 405 nm, and the kinetic parameters (i.e., the maximum reaction rate, V_max_) and the Michaelis–Menten constant, K_m_), were determined by plotting the double reciprocal of the Lineweaver–Burk equation, where V is the enzyme reaction rate and [S] is the concentration of the substrate.

##### 2.6.4.3 Inhibition of α-amylase

To determine the α-amylase inhibition activities of the SDF samples, the method described by Sangilimuthu et al. [42] was employed with slight modifications. A 2.0 U/mL pig pancreatic α-amylase solution was prepared using 0.1 M phosphate buffer. A 1 % solution of 1 % soluble starch was also prepared. For sample preparation, the sample solution (40 μL) was mixed with the α-amylase solution (50 μL), and allowed to react at 37 ℃ for 25 min. Subsequently, the soluble starch solution (50 μL) was added, and the reaction was allowed to continue for 15 min. After this time, 3,5-dinitrosalicylic acid (160 μL) was added and the mixture was allowed to stand for 5 min before boiling. Consequently, the mixture was diluted with distilled water to twice the volume and an aliquot (200 μL) was extracted for analysis. To prepare the blank sample, the enzyme solution was replaced with an equal volume of buffer, whereas to prepare the control, the sample was replaced with an equal volume of ddH_2_O. To prepare the blank control, the sample was replaced with an equal volume of ddH_2_O and enzyme solution in buffer. The absorbance was measured at 405 nm, acarbose was used as a positive control, and the enzyme activity inhibition rate was calculated as described in Section 2.4.6.1.

##### 2.6.4.4 Dynamic analysis of α-amylase inhibition

To evaluate the α-amylase inhibition activities of the SDF samples, the method reported by Xu et al. [43], [75] was followed with slight modifications. The relevant dynamic parameters for α-amylase inhibition were determined for the various samples. Initially, using an α-glucosidase concentration of 0.5 U/mL, the sample solution (40 μL; different concentrations) was added to the 96-well plate along with α-amylase (50 μL). After heating at 37 ℃ for 15 min, the soluble starch solution (50 μL) was added, and the reaction was continued at 37 ℃ for 15 min. The addition of 3,5-dinitrosalicylic acid (160 μL) followed and the mixture was allowed to stand for 5 min before boiling. The mixture was then diluted using distilled water to twice the volume and an aliquot (200 μL) was extracted for analysis. The absorbance was measured at 405 nm, and the kinetic parameters (i.e., the maximum reaction rate, V_max_) and the Michaelis–Menten constant, K_m_), were determined by plotting the double reciprocal of the Lineweaver–Burk equation, where V is the enzyme reaction rate, and [S] is the concentration of the substrate.

### 2.7 Statistical analysis

All experiments were performed at least in triplicate, and the data are expressed as the mean ± standard deviation (SD). SPSS26.0 (SPSS Inc., USA), MestReNova (version 15.0.1, Spain), ChemDraw (version 18.0, USA), and Origin 2023 (USA) software were employed during the course of this work. The differences between the groups were compared using the least significant difference (LSD) and *t*-test.

## 3 Results and discussion

### 3.1 Yield and compositional analysis of the rice husk SDF and IDF

SDFs obtained using the four processing/treatment methods showed significant differences in yields, as outlined in Table 1. More specifically, the yields decreased in the order of CE-SDF (18.64 ± 0.16 %) > C-SDF (12.56 ± 0.84 %) > E-SDF (6.96 ± 0.13 %) > N-SDF (2.13 ± 0.07 %). Compared to the non-treated sample, hydrodynamic cavitation treatment and hydrodynamic cavitation combined with enzymatic treatment led to yield increases of 5.897 and 8.751 times, respectively. Based on the yield of the remaining IDF, the obtained results suggested that hydrodynamic cavitation and enzymatic treatment promoted the decomposition of IDF, induced depolymerization, and significantly improved the SDF yield. Notably, hydrodynamic cavitation can generate local high-concentration free radicals through the collapse of air bubbles, whilst also forming enhanced dissociation forces through the “water phase combustion” effect, which can break the covalent bonds between cellulose and hemicellulose and increase the SDF content [44]. The active enzymes can further break the β-glycosidic bond between cellulose and hemicellulose, exposing additional hydrophilic groups and improving their hydration abilities [45]. Therefore, the combination of hydrodynamic cavitation and enzymatic treatment led to a significant increase in the SDF/IDF ratio. This shows that the synergistic effect of the hydrodynamic cavitation technology and snail enzyme can effectively destroy glycosidic bonds, release active ingredients, and significantly promote the conversion of IDFs into SDFs [46], [55], [59]. Among all treatments, the hydrodynamic cavitation method combined with the snail enzyme treatment afforded the highest SDF yield. This may be attributed to the synergistic treatment conditions being more effective in destroying the internal structure of the cell wall, thus accelerating the conversion of insoluble components [47]. The porous structure formed after hydrodynamic cavitation combined with snail enzyme treatment further verified that SDF experienced significant erosion, showing significant differences compared with untreated N-SDF. In addition, the SDF yields of the hydrodynamic cavitation treatment combined with the snail enzyme treatment were significantly higher compared with the extraction levels of bamboo shoots (7.28 %) and grapefruit peel (3.74 %)[48], [49]. In addition, based on the component analysis results presented in Table 1, it was clear that the C-SDF and CE-SDF samples contained significantly reduced crude fat contents. The fat in rice husk mainly derives from the natural wax layer and endogenous lipids (such as triglycerides, fatty acids, and phospholipids), which are combined with the fiber matrix through hydrophobic interactions or hydrogen bonds. The mechanical force of hydrodynamic cavitation can destroy the fiber structure and release some lipids, while the enzymatic treatment may hydrolyze the relevant bonds and further promote lipid separation. The interactions between these lipids and fibers may affect the structural and functional modifications of the fibers. However, the variations in the moisture, protein, and ash contents were not particularly significant. The significant increase in the yield of the C-SDF samples is mainly attributed to the strong shear force, micro-jet, and bubble collapse effects generated during the hydrodynamic cavitation treatment. These physical effects destroy the microstructure of the fiber (such as cell wall rupture and increased pores), thereby releasing more SDF. However, these physical damages mainly act on the fiber structure and intermolecular interactions (such as hydrogen bond breaking) rather than causing significant chemical bond breaking (such as glycosidic bond). Therefore, no obvious chemical bond breaking characteristics are observed in the FT-IR spectra.

**Table 1:** SDF and IDF yields following extraction using the different approaches. The chemical compositions, particle distributions, and physicochemical properties are also shown for SDFs.

Property/characteristic		N-SDF	E-SDF	C-SDF	CE-SDF
IDF and SDF yields (%)	SDF (%)	2.13 ± 0.07^c^	6.96 ± 0.13^c^	12.56 ± 0.84^b^	18.64 ± 0.16^a^
	IDF (%)	83.61 ± 0.77^a^	78.78 ± 1.02^a^	73.18 ± 0.29^b^	67.1 ± 0.11^c^
	TDF (%)	85.74 ± 0.09^a^	85.74 ± 0.04^a^	85.74 ± 0.01^a^	85.74 ± 0.12^a^
Chemical compositions of the SDFs (%)	Fat (%)	1.08 ± 0.04^a^	0.09 ± 0.01^a^	0.08 ± 0.03^b^	0.06 ± 0.04^c^
	Ash (%)	2.07 ± 0.12^a^	2.13 ± 0.08^a^	2.08 ± 0.16^a^	2.19 ± 0.04^a^
	Protein (%)	0.05 ± 0.01^c^	0.07 ± 0.03^b^	0.05 ± 0.01^b^	0.04 ± 0.01^a^
	Moisture (%)	2.26 ± 0.06^a^	2.08 ± 0.13^a^	2.28 ± 0.16^a^	2.34 ± 0.11^a^
Particle distributions of the SDFs	D_10_ (nm)	198.33 ± 0.21^a^	167.58 ± 0.18^b^	115.33 ± 0.23^c^	104.03 ± 0.08^c^
	D_50_ (nm)	280.67 ± 0.11^a^	195.33 ± 0.12^b^	128 ± 0.11^c^	122.33 ± 0.26^c^
	D_90_ (nm)	399 ± 0.13^a^	328.67 ± 0.46^b^	198.67 ± 0.18^c^	190.33 ± 0.04^c^
	PDI	0.273 ± 0.021^a^	0.268 ± 0.003^a^	0.139 ± 0.016^b^	0.103 ± 0.003^c^
	Specific surface area (m^2^/g)	0.726 ± 0.021^a^	0.758 ± 0.001^a^	1.216 ± 0.008^b^	1.718 ± 0.002^c^
	Zeta value (mV)	−16.39 ± 0.28^a^	−16.48 ± 0.16^a^	−33.27 ± 0.09^b^	−36.39 ± 0.12^c^
Physicochemical properties of the SDFs	WHC (g/g)	2.07 ± 0.02^c^	3.58 ± 0.18^a^	5.24 ± 0.11^b^	8.52 ± 0.09^d^
	OHC (g/g)	2.38 ± 0.12^a^	2.06 ± 0.64^a^	3.16 ± 0.17^b^	4.85 ± 0.29^c^
	SC (mL/g)	3.87 ± 0.15^d^	4.07 ± 0.17^c^	6.28 ± 0.24^b^	7.29 ± 0.25^a^
Molecular weight	M_n_ (kDa)	2.992	2.287	2.155	1.679
	M_w_ (kDa)	159.52	143.729	104.933	66.185
	M_w_/M_n_	53.315	62.846	48.693	39.419

Note: The letters indicate values in the same column that differ significantly (*p* < 0.05; one-way ANOVA followed by Duncan’s test).

Abbreviations: TDF, Total Dietary Fiber; IDF, Insoluble Dietary Fiber; SDF, Soluble Dietary Fiber.

### 3.2 Particle size distributions and zeta potential measurements

The four treatment methods significantly affected the particle size distributions of the SDF samples, as presented in Table 1, where the D_10_, D_50_, and D_90_ parameters are given (i.e., the sizes below which 10, 50, and 90 % of all particles are found based on the cumulative particle size distribution). The untreated N-SDF exhibited the largest particle size, followed by the E-SDF, C-SDF, and CE-SDF samples. In addition, the specific surface areas of the various samples increased with a decrease in the particle size, indicating that treatment has a positive effect on reducing the particle size. This reduction in the particle size is closely related to the application of both hydrodynamic cavitation and enzymatic treatment. More specifically, during hydrodynamic cavitation, the rice husk powder is affected by cavitation and severe vibrations, resulting in the formation of tiny bubbles inside the particles in the liquid. These bubbles roll into a jet as the liquid is rotated at high speeds, gradually increasing in volume. When the bubbles grow to a certain size, they collide violently with the inner walls of the instrument, causing the bubbles to burst. This rupture process generates high pressure microjets, forming high-energy shock waves that gradually break down the dietary fiber structure, as described by Peng et al. [22]. Similarly, the snail enzymes degrade IDFs by breaking the hydrogen bonds between the molecular chains and reducing the degree of polymerization. This leads to the smallest particle size being observed for the CE-SDF sample. Moreover, the polydispersity indices resulting from the other treatment approaches were higher than that of CE-SDF, indicating that the distribution of the CE-SDF particles in an aqueous solution is more uniform [38], [50].

Zeta potential (ζ-potential) refers to the potential of the particle shear plane. It is an important indicator of the stability of colloidal dispersions and is often used to describe the electrostatic interaction between colloidal particles. Generally speaking, when the absolute value of the zeta potential is less than 30 mV, the system is unstable and the particles are prone to aggregation [51]. Zeta potential analysis revealed the surface charge characteristics of SDFs, which are closely related to their ability to form colloids [52]. More specifically, the zeta potentials of the N-SDF, E-SDF, C-SDF, and CE-SDF samples were − 16.39 ± 0.28, −16.48 ± 0.16, −33.27 ± 0.09, and − 36.39 ± 0.12 mV, respectively. Notably, the absolute zeta potentials of the C-SDF and CE-SDF samples exceeded 30 mV, indicating their potentially excellent colloidal stabilities. As previously reported, SDFs possess abundant negative charges, partake in strong electrostatic attractions, and exhibit a strong gelling ability [53]. Feng et al. [54] showed that the strong electrostatic force between the alkaline hydrogen peroxide-modified SDF and Ca^2+^ may be another important reason for the gelation of M−SDF. The lower the negative charge of the modified SDF, the greater its dissociation degree, which can produce stronger electrostatic attraction with other ions, and may form a stronger gel in the presence of Ca^2+^ [46], [55], [59], [56]. Therefore, obtained results suggest that both C-SDF and CE-SDF will exhibit good solution stabilities and gelling abilities.

The results of the four treatment methods show that N-SDF (untreated group) had the lowest SDF yield and unchanged chemical structure, while hydrodynamic cavitation (C-SDF) and enzymatic treatment (E-SDF) increased the SDF yield to varying degrees. The synergistically-treated CE-SDF sample showed the highest SDF yield and significant structural modification, indicating that the synergy of the two methods maximized the fiber modification effect.

### 3.3 Analysis of the functional characteristics

As outlined in Table 1, the WHC, OHC, and SC values of SDFs subjected to hydrodynamic cavitation were higher than those of N-SDF, with the CE-SDF sample exhibiting the highest values. This may be due to the hydrodynamic cavitation rendering the microstructure of SDF loose and porous, which facilitates the further degradation by the snail enzymes, exposing a greater number of hydrophilic and lipophilic groups, and effectively enhancing the above characteristics.

### 3.4 Structural analysis of the rice husk before and after SDF modification

#### 3.4.1 TEM analysis of the rice husk before and after SDF treatment

**Fig. 2:**
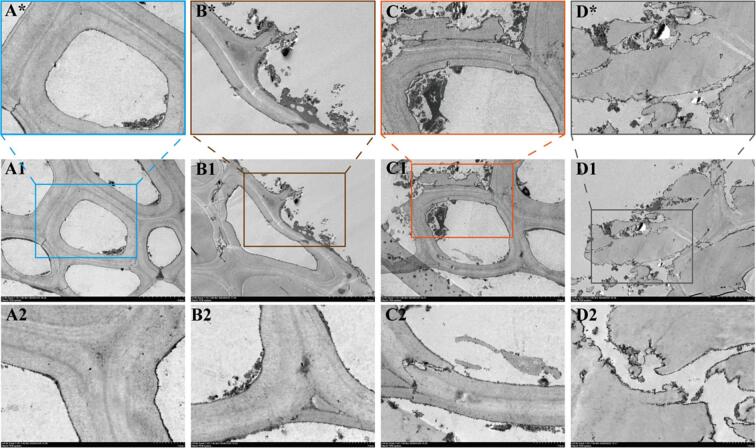
TEM images of the four SDF test groups. A, untreated rice husk fibers; B, fibers treated with snail enzymes; C, fibers treated with hydrodynamic cavitation; D, fibers treated with a combination of hydrodynamic cavitation and snail enzymes.

The cell structure of the untreated rice husk powder was initially examined using TEM. As shown in Fig. 2**A**, complete and stable cell walls can be observed with a tight fibrous texture. In addition, the cytoplasm is tightly wrapped within the cell wall, representing a good physical stability and a good integrity. The intact covalent bonds between the cellulose, hemicellulose, and lignin components indicate their strong ability to withstand external physical or chemical treatments. Consequently, further treatment is required to release SDFs. Following treatment with the snail enzymes alone, the local cell wall ruptured (Fig. 2**B**), the DF tissue began to disintegrate, and some cytoplasm overflowed. This indicates that the snail enzymes effectively destroyed certain key chemical bonds within the DF structure, including the β-glycosidic bond between cellulose and hemicellulose. Indeed, it is well known that enzymatic hydrolysis can selectively act on specific chemical bonds, leading to the relaxation and rupture of the DF structures. As the cell wall relaxes, some SDF in the rice husk powder is released, indicating that the enzymatic treatment can improve the extraction efficiency of SDFs. In addition, as shown in Fig. 2**C**, the rice husk powder treated with hydrodynamic cavitation alone, exhibited significant physical damage with local rupture and delamination of the DF structure, including the observation of newly formed voids in the cell wall. This was attributed to the collapse of air bubbles during hydrodynamic cavitation, which results in a significant destructive force, causes DF breakage, and increases the DF porosity. Owing to the formation of new pore structures during this process, the specific surface area of DF increased, which further promoted the release of SDF. These results indicate that hydrodynamic cavitation treatment efficiently promoted the physical damage of the DF structure. Furthermore, Fig. 2**D** shows the rice husk powder subjected to the combined hydrodynamic cavitation plus enzymatic treatment approach. It can be seen that the DF structure was almost destroyed, the cell wall was disintegrated, and the cytoplasm was scattered in the surrounding area; essentially, the original shape was lost. This was attributed to the synergistic effect between the powerful destructive force of hydrodynamic cavitation and the selective chemical degradation imparted by enzymatic treatment. It was therefore proposed that hydrodynamic cavitation initially relaxes and partially depolymerizes the dietary fiber structure, providing more opportunities for enzyme degradation. The high temperature, high pressure, and shock waves generated by hydrodynamic cavitation further enhance the degradation effect of the enzymes, promoting a more thorough disintegration of DF. Consequently, the cell wall was destroyed, and large amounts of SDF were released, demonstrating the enormous potential of the combined treatment method for SDF extraction.

#### 3.4.2 SEM analysis of the rice husk before and after SDF treatment

**Fig. 3:**
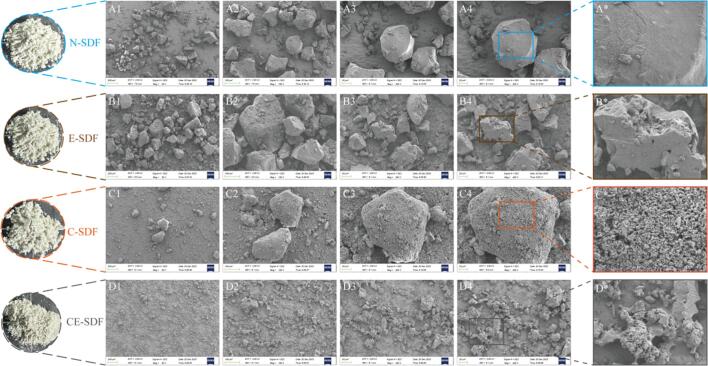
SEM images of the four SDF samples obtained using different approaches. A range of magnifications are provided: A1–D1 (50 × ), A2–D2 (150 × ), A3–D3 (300 × ), and A4–D4 (400 × ). Images of the N-SDF (A1, A2, A3, and A4), E-SDF (B1, B2, B3, and B4), C-SDF (C1, C2, C3, and C4), and CE-SDF (D1, D2, D3, and D4) samples. A* , B* , C* , and D* : Local amplifications.

The influence of the different treatment approaches on the microstructures of the SDF particles were subsequently analyzed using SEM, as presented in Fig. 3. More specifically, the untreated N-SDF appeared as dense, irregular block structures with smooth surfaces and no obvious grooves or voids (Fig. 3**A1–3A4**). In contrast, the SDF treated with snail enzymes (E-SDF) showed slight structural changes, with a rough and uneven surface and the appearance of voids and cavities (Fig. 3**B1–3B4**). This may be due to the enzymatic hydrolysis converting IDF into SDF, resulting in a decrease in the molecular weight. Furthermore, the SDF treated with hydrodynamic cavitation (C-SDF) showed a more obvious structural damage, with the overall shape transforming into a honeycomb-like structure with dense surface voids as well as loose and porous structures, thereby increasing the specific surface area (Fig. 3**C1–3C4**). Moreover, the combined action of the hydrodynamic cavitation and snail enzyme treatment (CE-SDF) led to the most thorough structural degradation, with the particle morphology changing from clumpy to fragmented. Furthermore, the particle size and number of particles increased, while the overall structure became looser (Fig. 3**D1–3D4**), as described above. These results confirmed the synergistic effect between the hydrodynamic cavitation and enzymatic treatment. It was also observed that the originally compact structure of the particles gradually exhibited signs of fracture, which is consistent with previous observations [57]. It was therefore concluded that this synergistic effect led to disruption of the cellulose and lignin matrix, thereby promoting the conversion of IDF to SDF [58].

#### 3.4.3 FT-IR analysis of the rice husk before and after SDF treatment

**Fig. 4:**
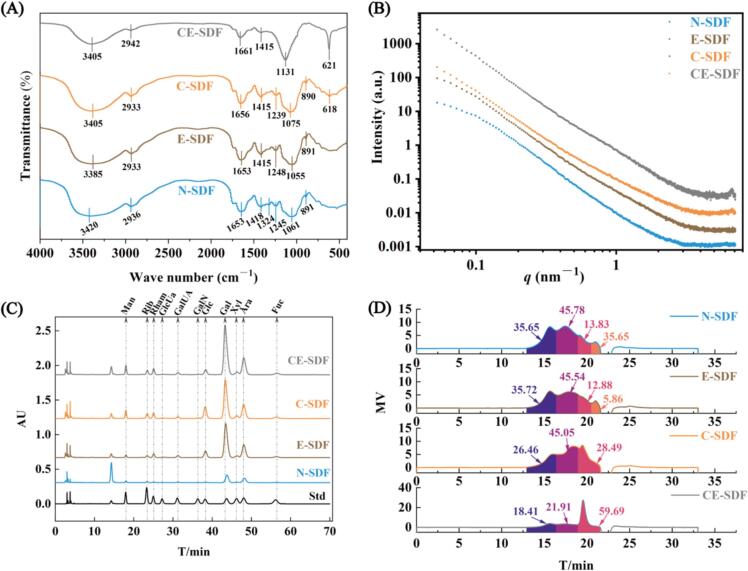
Results for the four SDF samples obtained using different analytical approaches. A: FT-IR, B: SAXS, C: HPLC, and D: GPC results.

FT-IR is widely used to study the functional group compositions and bonding configurations of compounds to reveal subtle differences in their molecular structures. As shown in Fig. 4**A**, the various SDF samples exhibited similar spectral characteristics, although some peak intensities varied between samples. More specifically, the FR-IR spectrum of N-SDF showed an absorption peak at 3420 cm^−1^ attributed to the O–H stretching vibrations in cellulose and hemicellulose. Owing to hydrogen bond formation between the hydroxyl groups, this peak is broad and strong, as reported by Liu et al. [30]. In E-SDF, the O-H stretching vibration peak shifts from 3420 cm^−1^ (N-SDF) to 3385 cm^−1^. This is due to the snail enzymes causing the cleavage of hydrogen bonds, which leads to a decrease in the vibration frequency of the O-H group, indicating a weakening of the strength of the intra- and intermolecular hydrogen bonds. Regarding C-SDF, the O–H peak shifted to 3405 cm^−1^, implying that the hydrodynamic cavitation disrupted some hydrogen bonds and altered the vibrational environment of the hydroxyl groups through strong shear forces and bubble collapse effects. In CE-SDF, although the synergistic effect of hydrodynamic cavitation and helicase also lead to the cleavage of hydrogen bonds in cellulose and hemicellulose, the strong physical effect of hydrodynamic cavitation significantly changed the overall structure of the fiber, possibly introducing new hydroxyl exposures or interference from other groups. This synergistic effect simultaneously changed the distribution and vibration environment of the O-H group, causing the masking or neutralization of the peak by other structural factors, resulting in no significant peak position shift. In terms of the C–H asymmetric stretching vibration peak of N-SDF at 2936 cm^−1^, this peak originates from the methyl and methylene groups of the sugars moieties. The corresponding peaks in the E-SDF and C-SDF samples (at 2933 cm^−1^) were not significantly different from that of the untreated sample, indicating that the local C–H bond structures did not change. However, the peak intensity was weakened for the CE-SDF sample (at 2942 cm^−1^), potentially due to partial breakage of the cellulose or non-cellulosic polysaccharide chains under the combined treatment. The weak absorption peak at 1750 cm^−1^ was attributed to the specific acetyl or C = O bonds of hemicellulose [60], while the peak at 1653 cm^−1^ originated from the C = O bond of the uronic acid ester group, as reported by Li et al. [61]. Moreover, the peak at 1415 cm^−1^ was attributed to the deformation vibration of the uronic acid carbonyl group [62], thereby indicating that all four SDFs are acidic polysaccharides with uronic acid structures. Additionally, the peak at 1324 cm^−1^ was attributed to the C–H angular vibration, as described by Wang et al. [63], whereas the peak corresponding to the C–O methoxy vibrations in lignin and hemicellulose was observed at 1245 cm^−1^ [64]. The peaks originating from the C–O, C–C, and C–O–C stretching vibrations in hemicellulose were observed at 1061, 1055, 1075, and 1131 cm^−1^, which correspond to the peak values of N-SDF, E-SDF, C-SDF, and CE-SDF, respectively [65]. Bending vibrations corresponding to β-glycosidic C–H bonds were also observed at 891 and 890 cm^−1^, indicating that the glycosidic bonds in the four SDFs were mainly in the β-configuration [66]. Finally, the peaks at 618 and 621 cm^−1^ were attributed to the variable angular vibration absorptions of the polysaccharide vertical β-C–H bonds [67].

Overall, the FT-IR results showed that the different treatment methods resulted in varying degrees of changes in the chemical structure of DF. N-SDF exhibited a complete DF structure and typical polysaccharide characteristics. The spectral changes in E-SDF were slight, indicating that the enzymatic hydrolysis treatment had little effect on the sample structure. However, after undergoing hydrodynamic cavitation treatment, more significant changes were observed for the C-SDF structure, especially in terms of molecular skeleton reorganization. Moreover, after hydrodynamic cavitation and enzymatic hydrolysis, significant degradation was observed for the CE-SDF sample, with the generation of abundant short chains and monosaccharides, and severe disruption of the original DF skeleton. These results confirm that hydrodynamic cavitation and enzymatic hydrolysis play a significant role in DF modification. In the CE-SDF sample, FT-IR analysis showed significant degradation phenomena, which are specifically reflected in the following aspects: The shift of the O–H stretching vibration peak indicates that the hydrogen bonding between or within the molecules is reduced. This change is due to the enzymatic treatment initiating hydrolysis of the polysaccharide chains, disrupting the hydrogen bond network in cellulose or hemicellulose. The decrease in the intensity of the C–H peak indicates that the glycosidic linkages were partially broken. This suggests that the enzymatic treatment acts on the chemical structure of cellulose or hemicellulose, causing the polysaccharide chains to brake into smaller molecules. The decrease in the peak intensity at 1750 and 1653 cm^−1^ indicates the degradation of specific structures (e.g., carbonyl groups) in hemicellulose and cellulose. These changes further support the conclusion that the cellulose and hemicellulose structures are significantly disrupted.

#### 3.4.4 SAXS analysis of the rice husk before and after SDF treatment

The relationship between the scattering intensity and the scattering vector *q* of SDF was subsequently investigated for the various samples (Fig. 4**B**). According to the scattering curves recorded for the four differently-processed SDFs (N-SDF, E-SDF, C-SDF, and CE-SDF), it was evident that the scattering intensity of N-SDF was low throughout the entire *q* interval, and the curve decreased rapidly, indicating that its structure was relatively large and its density was low. This was attributed to the relatively complete structure of SDF and its high degree of molecular aggregation, which resulted in a weaker intensity under SAXS conditions. The scattering intensity of E-SDF was slightly higher than that of N-SDF, and the curve showed a slower decrease at lower *q* values. This increase was attributed to the enzymatic hydrolysis effectively decomposing some DF structures, releasing smaller fragments and resulting in a more compact microstructure. Thus, although enzymatic treatment led to DF depolymerization, it also allowed retention of a certain molecular aggregation state. Regarding C-SDF, the observed scattering intensity was higher than those of the N-SDF and E-SDF samples, and the curve showed a significantly slower decrease in the larger *q* range. Therefore, the hydrodynamic cavitation treatment significantly changed the DF microstructure through physical destruction, producing abundant small molecular fragments and pores, increasing the surface area of DF, and increasing the number of intermolecular interactions. The highest scattering intensity was observed for the CE-SDF sample, especially at low *q* values, showing a slow downward trend and the smoothest curve. This combined processing approach employs the synergistic effects of hydrodynamic cavitation and enzymatic hydrolysis to thoroughly destroy the DF structure, resulting in a large number of small molecular fragments and highly depolymerized structures. The microstructure of CE-SDF was the most compact and uniform, thereby accounting for the strongest scattering intensity under SAXS conditions. Such results indicate that this method extracted the highest quantity of water-soluble DF components from the rice husk.

#### 3.4.5 HPLC analysis of the rice husk before and after SDF treatment

The chromatograms of the four SDF monosaccharides are shown in Fig. 4**C**, and the results are summarized in Table 2. As indicated, the four SDF contain mannose glucoside, nuclear glucose, rhamnose, glucuronic acid, galacturonic acid, glucose, galactose, xylose, arabinose, and fucose. This result was consistent with the near-infrared spectroscopy results, indicating the presence of galactose, xylose, and arabinose. More specifically, the main monosaccharides present in N-SDF were identified as glucose (8.93 ± 0.23 %), galactose (53.74 ± 0.01 %), and arabinose (21.73 ± 0.11 %), which is consistent with the results of Huang and Ma [68] and Yang et al. [69]. Similarly, the main monosaccharides in E-SDF were glucose (12.21 ± 0.01 %), galactose (48.35 ± 0.14 %), and arabinose (18.69 ± 0.02 %). In contrast, the main monosaccharides in C-SDF were galactose (56.02 ± 0.21 %) and arabinose (20.09 ± 0.12 %), similar to the case of CE-SDF, wherein the main monosaccharides were galactose (48.59 ± 0.02 %) and arabinose (31.23 ± 0.16 %).

**Table 2:** Monosaccharide compositions of SDFs extracted using the different approaches (normalized content, %).

Monosaccharide	N-SDF	E-SDF	C-SDF	CE-SDF
Mannose	1.77	4.25	4.27	4.78
Rhamnose	3.41	3.92	3.72	2.01
Glucuronic acid	0.44	0.63	0.39	0.35
Galacturonic acid	2.50	1.78	1.84	3.28
Glucose	8.93	12.21	4.73	1.84
Galactose	53.74	48.35	56.02	48.59
Xylose	2.81	3.22	3.04	2.91
Arabinose	21.73	18.69	20.09	31.23
Fucose	2.23	2.64	2.38	0.94

Qi et al. reported that the arabinose content significantly increased after the destruction of hemicellulose [70]. Thus, the current observation that the CE-SDF sample exhibited the highest arabinose content indicates that the combined effect of hydrodynamic cavitation and enzymatic hydrolysis leads to the degradation of hemicellulose. In addition, the galacturonic acid content was relatively high in CE-SDF, potentially due to the hollowing of the DF structure during hydrodynamic cavitation, and the subsequent enzyme degradation generating galacturonic acid. Previous studies have shown that higher levels of glucuronic acid are associated with a stronger ability to scavenge hydroxyl radicals [71], leading to enhanced antioxidant effects [72]. It was therefore speculated that CE-SDF may exhibit good antioxidant properties.

#### 3.4.6 GPC analysis of the rice husk before and after SDF treatment

The GPC analysis of the molecular weight distributions of the four SDF samples before 25 min (Fig. 4**D**) showed that both N-SDF and E-SDF exhibited four main component peaks, whereas C-SDF and CE-SDF produced only three main peaks. In the case of N-SDF, the first peak appeared at a retention time of 15.66 min, with a peak area of 35.65 % and a molecular weight of 188.033 kDa. The second peak appeared at 17.447 min, with an area of 45.78 % and a molecular weight of 11.302 kDa, while the third peak was observed at a retention time of 18.95 min, with an area of 13.83 % and a molecular weight of 1.147 kDa. The fourth peak appeared at 20.988 min, with an area of 4.73 % and a molecular weight of 0.226 kDa. Regarding E-SDF, the first peak appeared at a retention time of 15.676 min, with a peak area of 35.72 % and a molecular weight of 162.747 kDa. The second peak appeared at 17.886 min, with an area of 45.54 % and a molecular weight of 7.361 kDa, while the third peak was observed at a retention time of 19.267 min, with an area of 12.88 % and a molecular weight of 0.94 kDa. The fourth peak appeared at 21.013 min, with an area of 5.86 % and a molecular weight of 0.226 kDa. The C-SDF sample exhibited the first peak at a retention time of 16.031 min, with a peak area of 26.46 % and a molecular weight of 136.391 kDa. The second peak was observed at 18.447 min, with an area of 45.05 % and a molecular weight of 7.165 kDa, while the third peak appeared at 19.467 min, with an area of 28.49 % and a molecular weight of 0.682 kDa. Moreover, for the CE-SDF sample, the first peak appeared at a retention time of 15.667 min, with a peak area 18.41 % and a molecular weight of 172.036 kDa, while the second peak was observed at 17.40 min, with an area of 21.91 % and a molecular weight of 11.644 kDa, and the third peak was detected at 19.542 min, with an area of 59.69 % and a molecular weight of 1.007 kDa.

The molecular weight distribution results for the four types of SDF were evaluated to determine the effects of the different treatment approaches (Table 2). The earlier the peak appears, the larger is the molecular weight. Thus, as outlined in the table, the following results were obtained: N-SDF (M_n_ = 2.992 × 10^3^ Da, M_w_ = 159.52 × 10^3^ Da, D = 53.315); E-SDF (M_n_ = 2.287 × 10^3^ Da, M_w_ = 143.729 × 10^3^ Da, D = 62.846); C-SDF (M_n_ = 2.155 × 10^3^ Da, M_w_ = 104.933 × 10^3^ Da, D = 48.693); and CE-SDF (M_n_ = 1.679 × 10^3^ Da, M_w_ = 66.185 × 10^3^ Da, D = 39.419).

Such results demonstrate that the proportion of the high-molecular-weight segments in N-SDF was 81.43 %, whereas the proportion of the low-molecular-weight segments was 18.56 %. After enzymatic hydrolysis to obtain the E-SDF sample, no significant changes were observed, i.e., the proportion of high-molecular-weight segments was 81.26 %, and the proportion of low-molecular-weight segments was 18.74 %. However, after treatment with hydrodynamic cavitation (C-SDF), the proportion of the high-molecular-weight segments decreased to 71.51 %, while the proportion of the low-molecular-weight segments increased to 28.49 %. Following combined hydrodynamic cavitation and enzymatic hydrolysis, the proportion of the high-molecular-weight segments in CE-SDF was reduced dramatically to 40.32 %, implying a conversion to low-molecular-weight segments (59.69 %). These results indicate that the structure loosened and fragmented, increasing the specific surface area to expose additional enzyme-binding sites, and promoting the generation of higher-molecular-weight segments.

In summary, the molecular weight distribution of N-SDF is relatively concentrated, mainly in the high molecular weight region. This indicates that the structure of N-SDF is intact and not affected by external physical or biological effects, while its internal molecular bonds are also not destroyed. Compared with N-SDF, E-SDF has obvious low molecular weight components and the main peak moves to the right (low molecular weight range); however, some high molecular weight components still exist. Treatment with snail enzymes resulted in a decrease in molecular weight, with the degradation process being relatively mild, affording both high and low molecular weight components. Cavitation is the explosion of microbubbles triggered by ultrasonic waves, instantly causing a high temperature, high pressure, and strong shear force, thus physically damaging the fiber structure. In C-SDF, cavitation broke the hydrogen bonds and hydrophobic interactions between the polysaccharide molecules, thereby loosening the structure; the generated shear force further destroyed the glycosidic bonds, especially the long-chain polysaccharides, resulting in a significant reduction in molecular weight. In CE-SDF, cavitation loosened the structure of DF, increased the exposure of the enzyme action sites, and improved the degradation efficiency of the snail enzymes. The high pressure and microturbulence generated by cavitation can promote the mixing of the enzymes and substrate as well as the reaction kinetics, thereby further accelerating the hydrolysis of the glycosidic bonds. In general, combined degradation uses the structural destruction effect of cavitation and the selective hydrolysis of snail enzymes to significantly improve the degradation efficiency, exhibiting a significant superimposed effect.

#### 3.4.7 NMR analysis of the rice husk before and after SDF treatment

**Fig. 5:**
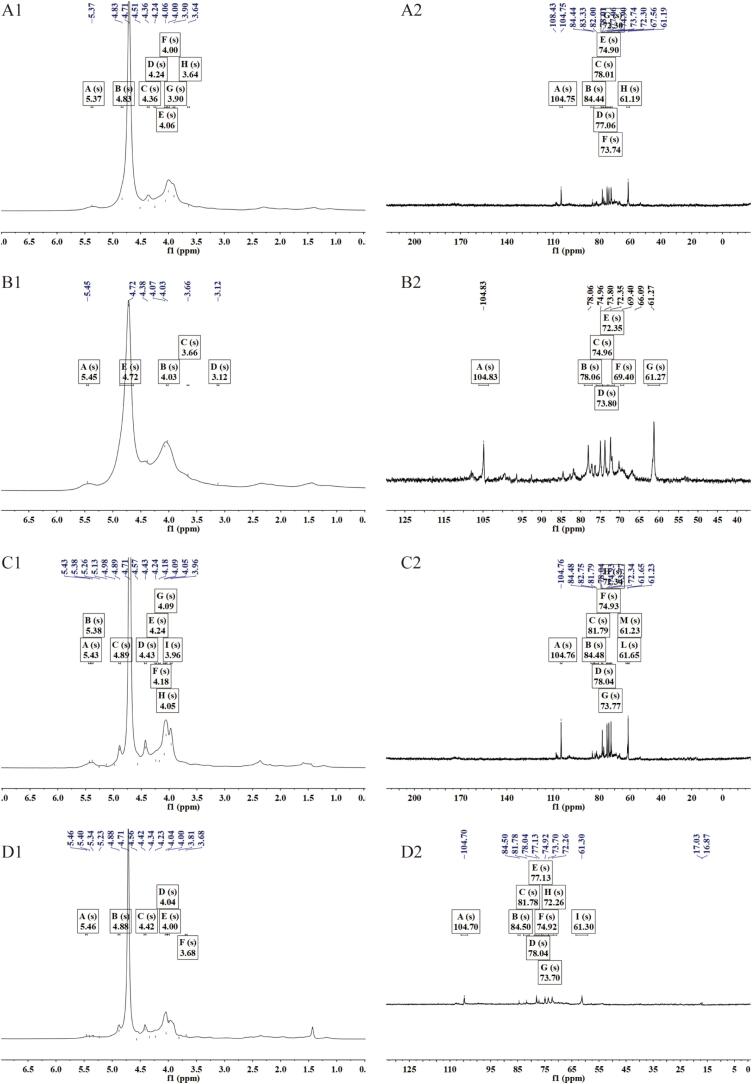
NMR spectra of the SDF samples obtained using different approaches. A1: ^1^ H NMR spectrum of N-SDF, B1: ^1^ H NMR spectrum of E-SDF, C1: ^1^ H NMR spectrum of C-SDF, and D1: ^1^ H NMR spectrum of CE-SDF^,^ A2: ^13^ C NMR spectrum of N-SDF, B2: ^13^ C NMR spectrum of E-SDF, C2: ^13^ C NMR spectrum of C-SDF, and D2: ^13^ C NMR spectrum of CE-SDF.

In the ^1^H NMR spectrum of the N-SDF sample, the peaks observed between *δ* 3.5 and 5.5 ppm correspond to the C–H and –OH groups in the sugar ring (Fig. 5**A1**). These signals originate from the glucose units of the polysaccharides, which are connected via β-1,4-glycosidic bonds. The narrow and symmetrical peak shape indicates the regularity of the N-SDF structure, suggesting the presence of a relatively uniform proton environment. In the ^13^C spectrum of the N-SDF species, the signals observed between *δ* 60 and 105 ppm correspond to the C1–C6 carbon atoms in the glucose unit. The signal at *δ* 100–105 ppm was attributed to the C1 atom (β-1,4-glycosidic bond), reflecting the stability of the polysaccharide structure. Furthermore, the sharp and symmetrical peak shapes reflect the uniformity of the carbon environments, which is consistent with the structures of cellulose and other high-molecular-weight polysaccharides. The N-SDF structure appeared stable and unaffected by external factors, resulting in a regular chemical environment in the NMR spectrum.

The weak peaks observed in the range of *δ* 3.5–4.5 ppm were examined to reflect the structural changes of some polysaccharide chains after enzymatic hydrolysis. As shown in Fig. 5**B1**, the signals corresponding to the hydrogen atoms at the end of the sugar chains are enhanced. Compared with the N-SDF spectrum, the E-SDF spectrum exhibited more complex peak shapes in this region, indicating the generation of more terminal groups and short-chain oligosaccharides. The peak width also increased, indicating that the enzymatic hydrolysis led to the heterogeneity of the proton environment and the formation of new short-chain oligosaccharides. A slight reduction in the peak area indicated the partial degradation of long-chain polysaccharides under enzymatic hydrolysis. Regarding E-SDF, the C1 signal at *δ* 100–105 ppm became more dispersed, reflecting partial cleavage of the glycosidic bonds (Fig. 5**B2**). Other peaks also showed slight changes in their chemical shifts, indicating that enzymatic hydrolysis altered the environments of some carbon atoms. A broadening of the peak shape and expansion of some peaks indicated the formation of new oligosaccharide structures through enzymatic hydrolysis.

The C-SDF sample exhibited more peaks in the *δ* 3.0–4.5 ppm region, indicating that hydrodynamic cavitation triggered the formation of a large number of short chains and terminal groups in the molecule (Fig. 5**C1**). These new peaks originated from the oligosaccharides and monosaccharides produced by hydrolysis. Compared to the E-SDF spectrum, the peak shape after hydrodynamic cavitation treatment was wider and more complex, reflecting the high heterogeneity of the molecular structure. In addition, the increased peak area in the *δ* 3.5–4.5 ppm region indicated that more terminal protons were exposed, which is consistent with the production of a large number of short-chain fragments under hydrodynamic cavitation conditions. The formation of short-chain oligosaccharides was also evident by the observation of new signals in the *δ* 60–80 ppm range of the ^13^C spectrum, which corresponds to carbon atoms located at the ends of the broken chains (Fig. 5**C2**). These results indicated the formation of short-chain oligosaccharides with irregular structures. Compared to enzymatic hydrolysis, hydrodynamic cavitation led to more complex peak shapes, suggesting that the degree of structural damage was more extensive.

Regarding the CE-SDF sample, a large number of complex new peaks appeared in the *δ* 3.0–4.0 ppm region, indicating that the combination of hydrodynamic cavitation and enzymatic hydrolysis produced highly degraded short-chain fragments, including a large number of oligosaccharides and monosaccharides (Fig. 5**D1**). Highly overlapping and expanding signals were evident, suggesting a heterogeneous proton environment, which is consistent with diversification of the molecular chains after degradation. The significant increase in the peak area, especially in the low chemical shift region, reflects the presence of more abundant protons in the short chains and terminal groups. In the ^13^C spectrum, numerous new peaks were observed in the *δ* 60–80 ppm region, indicating the formation of various short chains and terminal groups of different lengths after the combined treatment (Fig. 5**D2**). The complexity of these signals indicated that the sugar chain was thoroughly degraded. Moreover, wide and complex signals were observed, suggesting a significant increase in structural heterogeneity and extremely diverse chemical environments for the carbon atoms.

### 3.5 Physical properties, chemical properties, and functional characteristics of the rice husk before and after SDF treatment

**Fig. 6:**
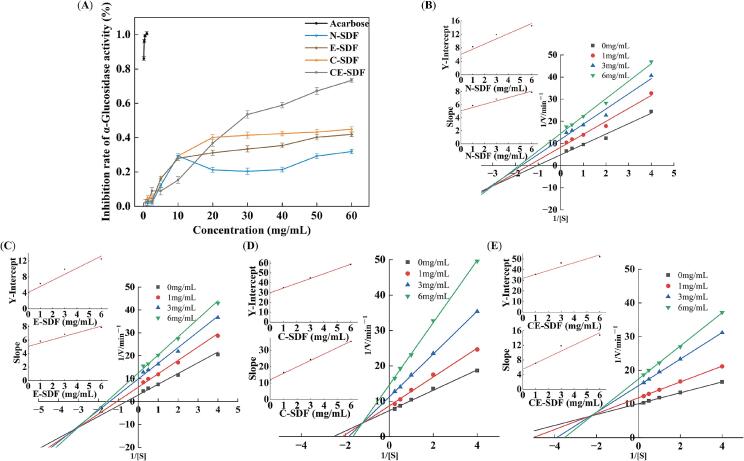
Inhibitory effects of the four SDF samples on α-glucosidase activity. The corresponding Lineweaver–Burk plots are also shown.

**Fig. 7:**
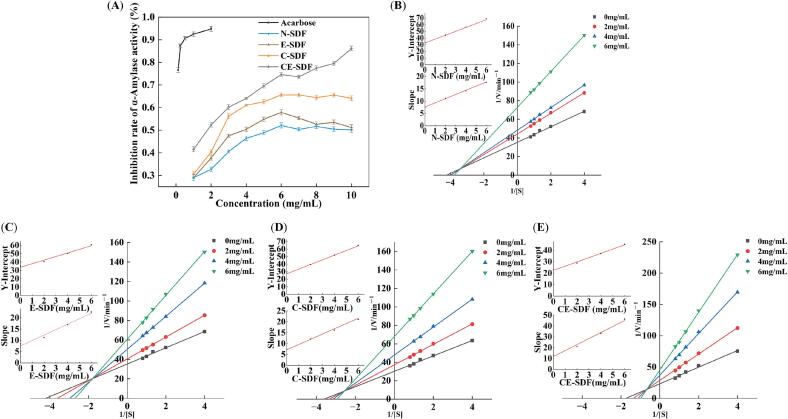
Inhibitory effects of the four SDF samples on α-amylase activity. The corresponding Lineweaver–Burk plots are also shown.

α-Glucosidase and α-amylase inhibitors are known to effectively reduce carbohydrate breakdown in the small intestine and slow the rate of blood glucose elevation [73]. Using pNPG as a substrate, the inhibitory effects of the four SDFs on α-glucosidase and α-amylase were evaluated *in vitro* (Fig. 6**A and 7A**). Using acarbose as a positive control, it was found that all four SDFs exhibited superior inhibitory effects to acarbose, in addition to demonstrating a dose-dependent relationship. The IC_50_ values for α-glucosidase inhibition were 22.523, 7.996, 5.748, and 2.893 mg/mL for N-SDF, E-SDF, C-SDF, and CE-SDF, respectively, while those for α-amylase inhibition were 7.025, 4.983, 2.794, and 1.727 mg/mL, respectively. These results indicate that at the same concentration, hydrodynamic cavitation combined with snail enzyme treatment produced SDFs with significantly enhanced inhibitory activities against both enzymes. Based on the results presented in the previous subsections, it is likely that N-SDF exhibited a poor enzyme binding ability owing to its large molecular weight and compact structure and minimal inhibitory effects due to a lack of exposed active sites. Following snail enzyme treatment alone, it was possible to effectively break the long-chain polysaccharides in SDF and generate small fragments or oligosaccharides. This increased the contact area with the enzyme, exposed additional hydroxyl groups, and enhanced the inhibitory ability of the E-SDF sample. Regarding C-SDF, hydrodynamic cavitation destroyed the SDF molecular structure through molecular chain breakage and structural deformation. Therefore, this treatment increased the active surface area of SDF, enhanced its enzyme binding ability, and significantly improved its inhibitory effect. Upon combining both hydrodynamic cavitation and enzymatic treatment, the molecular structure of SDF was altered to the greatest extent, producing smaller and more dispersed active fragments. The hydroxyl groups of SDF were more exposed, which greatly enhanced their ability to bind to enzymes. Consequently, CE-SDF exhibited the most significant inhibitory effect on the activities of α-glucosidase and α-amylase. This indicates that the inhibition rate of the enzymatic activity is closely related to the reduction of molecular weight. The reduction in molecular weight helps to expose more enzyme active sites, thereby enhancing the binding of SDF to the enzyme and leading to enhanced inhibition [74], [43], [75].

The α-glucosidase inhibition behaviors of the four SDFs were analyzed using their corresponding Lineweaver–Burk curves. From the double reciprocal plots of the enzymatic reaction rates at different SDF mass concentrations (Fig. 6**B–6E**), it was evident that K_m_ gradually decreased and V_max_ gradually decreased as the mass concentrations of N-SDF and E-SDF increased. The various curves intersected in the third quadrant, indicating that N-SDF and E-SDF were mixed anti-competitive inhibitors of α-glucosidase, only inhibiting the enzymatic reaction upon binding to the enzyme–substrate complex [76]. Similarly, K_m_ and V_max_ gradually increased and decreased, respectively, as the mass concentrations of C-SDFs and CE-SDF increased. However, the curves intersected in the second quadrant, indicating that C-SDF and CE-SDF were competitive inhibitors of α-glucosidase, either binding to free α-glucosidase or affecting the enzymatic reactions through binding to the enzyme–substrate complex. According to the data presented in Table 3, the K_i_ values obtained for the four SDFs were K_i(N-SDF)_ = 101.429, K_i(E-SDF)_ = 101.429, K_i(C-SDF)_ = 61.589, and K_i(CE-SDF)_ = 32.941. Thus, CE-SDF exhibited the highest affinity for enzyme binding, in addition to demonstrating the strongest inhibitory effects.

**Table 3:** Dynamic parameters for α-glucosidase inhibition by the four SDFs.

SDF	Concentration (mg/mL)	Michaelis–Menten equation	K_m_ (mg/mL)	V_max_ (mg/mL·min)	K_i_ (mg/mL)	K_i_^'^ (mg/mL)
N-SDF	0	y = 4.69x + 4.88	0.961	0.205	101.429	39.797
	10	y = 5.85x + 8.30	0.702	0.120		
	30	y = 6.86x + 11.89	0.576	0.084		
	60	y = 7.88x + 14.50	0.544	0.069		
E-SDF	0	y = 4.69x + 2.88	1.627	0.347	101.429	26.695
	10	y = 5.85x + 6.30	0.930	0.159		
	30	y = 6.86x + 9.89	0.693	0.101		
	60	y = 7.88x + 12.50	0.630	0.080		
C-SDF	0	y = 11.52x + 29.32	0.392	0.034	61.589	30.790
	10	y = 16.31x + 34.77	0.473	0.029		
	30	y = 24.25x + 44.85	0.534	0.022		
	60	y = 35.23x + 58.44	0.599	0.017		
CE-SDF	0	y = 4.84x + 30.28	0.160	0.033	32.941	87.284
	10	y = 7.05x + 35.52	0.197	0.028		
	30	y = 11.83x + 46.31	0.260	0.022		
	60	y = 14.75x + 52.19	0.277	0.019		

Finally, the α-amylase inhibition behaviors were evaluated in a similar manner. The double reciprocal plots of the enzymatic reaction rates at different SDF mass concentrations (Fig. 7**B–7E**) show that K_m_ and V_max_ gradually decreased as the mass concentrations of the four SDFs were increased. The curves intersected in the second quadrant, and C-SDF and CE-SDF were identified as competitive inhibitors of α-amylase. Consequently, these species could either bind to free α-amylase or influence the enzymatic reactions through binding to the enzyme–substrate complex. According to the data presented in Table 4, the K_i_ values for the four SDFs were K_i(N-SDF)_ = 4.487, K_i(E-SDF)_ = 3.113, K_i(C-SDF)_ = 2.980, and K_i(CE-SDF)_ = 2.128. Therefore, CE-SDF exhibited the highest affinity for enzyme binding, in addition to demonstrating the strongest inhibitory effects.

**Table 4:** Dynamic parameters for α-amylase inhibition by the four SDFs.

SDF	Concentration (mg/mL)	Michaelis–Menten equation	K_m_ (mg/mL)	V_max_ (mg/mL·min)	K_i_ (mg/mL)	K_i_^'^ (mg/mL)
N-SDF	0	y = 8.35x + 35.24	0.234	0.028	4.487	5.604
	2	y = 11.09x + 44.23	0.255	0.023		
	4	y = 12.13x + 48.24	0.255	0.021		
	6	y = 19.34x + 72.73	0.271	0.014		
E-SDF	0	y = 8.35x + 35.24	0.234	0.028	3.113	7.839
	2	y = 11.20x + 40.53	0.280	0.025		
	4	y = 16.97x + 50.31	0.339	0.020		
	6	y = 22.53x + 60.70	0.360	0.016		
C-SDF	0	y = 8.35x + 30.24	0.276	0.033	2.980	4.410
	2	y = 11.09x + 37.23	0.299	0.027		
	4	y = 15.15x + 47.81	0.318	0.021		
	6	y = 23.07x + 67.91	0.346	0.015		
CE-SDF	0	y = 13.28x + 23.23	0.571	0.043	2.128	6.007
	2	y = 21.01x + 28.82	0.735	0.035		
	4	y = 33.24x + 37.18	0.897	0.027		
	6	y = 46.15x + 45.35	1.015	0.022		

### 3.6 Synergistic mechanism of hydrodynamic cavitation and snail enzyme

During the synergistic treatment, the mechanism of action of hydrodynamic cavitation and helicase shows a very significant synergistic effect. The synergistic mechanism of hydrodynamic cavitation and snail enzyme reflects the high complementarity of physical and biological processes. Hydrodynamic cavitation destroys the covalent bonds of cellulose and hemicellulose in rice husk fibers through the high temperature, high pressure, micro-jet, and strong shear force generated by cavitation collapse. This decreases the density of the fiber structure and forms a porous and loose state, while significantly reducing the crystallinity of the fiber. This physical destruction increases the specific surface area of the fiber, exposing more reaction sites and providing more degradation space for snail enzymes. Snail enzymes are complex enzymes exhibiting multiple enzymatic activities such as degrading cellulose, hemicellulose, and pectin. They decompose macromolecular fibers into short-chain oligosaccharides and monosaccharides by acting on specific chemical bonds such as β-glycosidic bonds. The synergistic treatment significantly changes the molecular structure and chemical properties of the fiber. FTIR and NMR results showed that the synergistic effect of the cavitation and enzymatic treatments further reduced the intermolecular hydrogen bonds in the fibers, lowered the degree of polymerization, and formed more hydrophilic functional groups.

In terms of functional properties, the CE-SDF obtained after the synergistic treatment of hydrodynamic cavitation and snail enzyme showed a higher WHC, OHC, and SC. This is because the fiber structure is loose and porous after the synergistic treatment, and the hydrophilic and lipophilic groups are fully exposed. In addition, the synergistic treatment significantly improved the biological activity of CE-SDF, exhibiting a strong competitive inhibition of α-glucosidase and α-amylase, with IC_50_ values of 2.893 and 1.727 mg/mL, respectively. Kinetic analysis showed that CE-SDF could bind to the enzyme by exposing more active sites, thereby enhancing the inhibition of the enzymatic activity. This synergistic treatment combines the efficient destructive power of physical treatment with the selective degradation of the enzymatic hydrolysis reaction, which not only maximizes the extraction efficiency of SDF from rice husk, but also significantly improves its structural and functional properties. Our findings provide a scientific basis and new ideas for the development of functional foods and hypoglycemic drugs.

## 4 Conclusion

In this study, four different processing methods were employed to extract SDFs from rice husk, and the effects of these treatments were evaluated in terms of the physicochemical properties, structural changes, and *in vitro* inhibitory effects of the extracted SDFs on α-glucosidase and α-amylase. Hydrodynamic cavitation combined with snail enzyme modification significantly improved the quality of SDF (i.e., CE-SDF). The highest yield was obtained for CE-SDF, and the particle size was effectively reduced, leading to an increased specific surface area and favorable zeta potential. In addition, WHC, OHC, and SC were maximized following this combined treatment approach. Following treatment, the SDF structure became loose and fragmented with changes in its functional groups. Consequently, abundant small-molecular fragments and highly depolymerized structures were generated, including numerous monosaccharides. Furthermore, the CE-SDF sample exhibited significant inhibitory effects on the activities of α-glucosidase and α-amylase, acting as a competitive inhibitor. These results indicate that the addition of CE-SDF to hypoglycemic drugs and functional foods could provide a new direction for slowing blood sugar elevation in patients.

## CRediT authorship contribution statement

**Zhigang Quan:** Writing – original draft, Software, Methodology, Formal analysis, Conceptualization. **Mingming Chen:** Writing – review & editing, Validation, Methodology, Conceptualization. **Dongjie Zhang:** Writing – review & editing, Supervision, Methodology, Funding acquisition, Conceptualization.

## Funding

This work was supported by the Basic Science Research Program through the National Program on Key Research Project (2023YFD1202705-6), Heilongjiang Province Specialty Discipline Project for the Production and Processing Advantages of Coarse Cereals (2022-78).

## Declaration of competing interest

The authors declare that they have no known competing financial interests or personal relationships that could have appeared to influence the work reported in this paper.

## Glossary

DF: dietary fiber
TDF: total dietary fiber
SDF: soluble dietary fiber
IDF: insoluble dietary fiber
CE-SDF: treated with hydrodynamic cavitation combined with snail enzyme hydrolysis
N-SDF: non-processed soluble dietary fiber
E-SDF: enzymatically treated soluble dietary fiber
C-SDF: hydrodynamic cavitation-treated soluble dietary fiber
TEM: Transmission electron microscopy
SEM: scanning electron microscopy
X-ray: Small-angle X-ray scattering
FT-IR: Fourier-transform infrared
HPLC: high-performance liquid chromatography
GPC: Gel permeation chromatography
NMR: Nuclear magnetic resonance

## Appendix A1

Based on the experimental equipment's processing power of 2.2 kW and processing volume of 5.0 L/h, it is preliminarily estimated that the total energy consumption of hydrodynamic cavitation treatment is about 0.55 kWh. The equipment's processing volume per hour is 5 L, so the energy consumption per liter of material is 0.11 kWh/L. This estimate provides a reference for subsequent process optimization and industrial application.
